# Prime Incision and Modified Moving Window: A Minimally Invasive Access for Breast Cancer Surgical Treatment

**Published:** 2016-09

**Authors:** Silvio E. Bromberg, Roberto Giordano

**Affiliations:** Hospital Israelita Albert Einstein Oncology Center, São Paulo, Brazil

**Keywords:** Breast cancer, Treatment, Minimally invasive surgery, Modified moving window, Prime Incision

## Abstract

**BACKGROUND:**

Conservative surgical treatment has been the treatment of choice for early breast cancer. It allows feasible oncological treatment with a satisfactory cosmetic approach and fast recovery. However, in some cases mastectomy is necessary. This study proposes a surgical approach with only one surgical access through the same incision, which is in line with precepts mentioned above. It is called the prime incision and modified moving window techniques.

**METHODS:**

Thirty one patients with a breast cancer diagnosis who would have to undergo surgery were enrolled. The proposed technique was used and its advantages and feasibility were assessed and evaluated.

**RESULTS:**

Twenty three conservative surgeries, 7 adenectomies and 1 axillary surgery were performed. All cases were appropriately treated and progressed without complications and had adequate aesthetic results. The technique presented allows only one surgical access to approach the axilla and breast cancer treatment.

**CONCLUSION:**

This study proved the aesthetic advantage provided by only one surgical incision access feasible for surgical treatment as a secure approach. The minimally invasive approaches of prime incision and modified moving window were shown to provide adequate surgical access with only one scar, thus having a better cosmetic result.

## INTRODUCTION

Conservative surgical treatment has been the treatment of choice for breast cancer. In cases in which the ratio of breast to tumor volume allows a feasible oncological resection, the conservative approach is better than mutilating and radical treatments because it is less invasive, is faster, and presents good aesthetic results.^1^^,^^2^ For patients requiring mastectomy, a variety of alternatives to the modified radical mastectomy exist, such as adenectomy with removal of the areolar-papillary complex – APC - (skin-sparing mastectomy) or adenectomy with preservation of the areolar papillary complex (nipple-sparing mastectomy).^1^^-^^5^ Aesthetic concerns made us more attentive to perform surgeries that involve a better aesthetic approach.

It is known that aesthetic success in surgical treatment of breast cancer treatment helps women achieve more suitable social and sexual reaclimation to normal life; however, lack of adequate oncological and cosmetic planning will lead to unsatisfactory aesthetic results, with unacceptable breast deformities. In order to achieve better oncological and aesthetic results, we developed a surgical approach by making only one small incision. This method can be used in both conservative surgeries and adenectomies. This surgical approach is more difficult because the surgery is performed through only one small incision, which restricts visibility and space for the resection; therefore, it requires greater expertise and skills on the part of the surgeon. In ideal conditions retractors must be used in conservative or radical surgical approaches, which may damage the flap as well as the incision edges, leading to a poor aesthetic result.^6^^,^^7^

In 2010, Masakuni Noguchi and Masashi Onikuchi described a technique called the moving window,^8^ in which a small periareolar incision was performed to make a dermo-cutaneous flap and a Alexis wound retractor was introduced (Applied Alexis wound retractor, Applied Medical Resources Co., Rancho Santa Margarita, CA, USA). The goal was to allow tissue mobility, provide good delimitation of the area to be resected, and protect the skin around the incision, ensuring satisfactory aesthetic results. It was suggested that for the axillary approach another incision should be made in the axilla following the same approach technique. Only for resections of segmentary lesions in the supero lateral quadrant was just one incision made for the breast and axillary approaches. Subsequent reports described the use of a wound retractor in the performance of periareolar adenectomies.^8^^-^^10^

The technique we proposed here combined the moving window surgical approach in only one incision. We used the wound retractor to facilitate the performance of only one incision in the breast lesion and axilla for both conservative surgeries and adenectomies**.** We named our technique the prime incision through the moving window approach, which we have modified; therefore, we called it the modified moving window.^8^^-^^10^ The objective of the present study was to assess and evaluate the use of the prime incision and modified moving window in relation to feasibility of a suitable surgery with free surgical edges as well as a suitable axillary approach showing the aesthetic results and complications.

## MATERIALS AND METHODS

Between January 2014 and March 2015, Israelita Albert Einstein Hospital Ethics Committee consented to the assessment of 31 patients with surgical indications for a breast cancer diagnosis that was confirmed by previous core biopsy. Patients in the study were aged 30 to 70 years and had breast cancer diagnosed through physical exam, imaging and pathological examination (percutaneous biopsy). The included patients had stage 0, I or II cancer (Table 1), with the indication for conservative surgical treatment or preserving adenectomy or APC or when it seemed feasible to perform this technique to treat local or loco-regional breast cancer recurrences. 

**Table 1:** Patients in the study with breast cancer diagnosed through physical exam, imaging and pathological examination (percutaneous biopsy

**Patient**	**Age**	**Stage**	**Location**	**Diagnostic**	**Surgery**
1	35	0	Multicent RIC	CDIS	NSM
2	53	II	QQLL	CDI	NSM
3	44	0	QSL	CDIS	Conservative
4	54	I	QQMM	CDI	Conservative
5	39	I	QSM	CDI	Conservative
6	47	I	QSM	CLI	Conservative
7	42	I	QSL	CDI	Conservative
8	38	II	QQSS	CDI	Conservative
9	47	II	QSL	CDI	SSM
10	43	I	QQII	CDI	Conservative
11	45	II	Interpecto RAL/ Level III	CDI	Axillar (Conservative )
12	46	II	QSL	CLI	SSM
13	60	I	QIM	CDI	Conservative
14	32	0	QSM	CDIS	Conservative
15	49	I	QSL	Tubular	Conservative
16	64	I	QSL	CDI	Conservative
17	52	I	QSL	CDI	Conservative
18	60	I	QQSS	CDI	Conservative
19	47	II	QSM	CDI	NSM
20	40	II	QSM	CLI	NSM
21	65	I	QIL	CDI	Conservative
22	70	I	QIL	CDI	Conservative
23	42	I	QQSS	CDI	Conservative
24	40	I	QSL	CDI	Conservative
25	61	II	QSM	CDI	Conservative
26	70	I	QQLL	CDI	Conservative
27	57	I	QQLL	CDI	Conservative
28	45	II	QSL	CDI	NSM
29	59	I	QSL	CDI	Conservative
30	57	II	QSL	CDI	Conservative
31	69	I	QIM	CDI	Conservative

CDIS ductal in situ carcinoma, QQMM intersection medial quadrants, CDI ductal invasive carcinoma, QQLL intersection lateral quadrants, NSM nipple sparing mastectomy, QSS intersection superior quadrants, SSM skin sparing mastectomy, QQII intersection inferior quadrants, QSL supero lateral quadrant, QSM medial supero quadrant, QIM medial infero quadrant

The criteria for wound retractor utilization as described by Noguchi and Onikuchi in conservative surgeries (breast size, areolar-papillary plate and tumor–breast relation) and adenectomies^8^^,^^9^ have been disregarded. The indication for wound retractor has been broadened to all conservative surgeries independently of tumor location, and for adenectomies with or without of preservation of APC**, **with incision through inframammary sulcus or peri-areolar. The use of wound retractor was indicated in all cases that have an indication for the prime incision technique (that is, use of only one incision). When the breasts were dense or there were any conflicting diagnoses between the imaging exams and physical exam, the selected patients who met the inclusion criteria were assessed through physical exam, mammography, breast ultrasonography, and magnetic resonance imaging (MRI) with contrast.

Systemic staging was done through thorax X-ray, abdominal ultrasonography, bone scintigraphy, and pre-operative exams. It is important to highlight that the proposed technique did not modify the treatment proposed to the patients and the date and type of surgery were kept as previously scheduled for adenectomy or conservative surgery. On the day of surgery (about 4 hours before the procedure), all patients received a peritumoral or subareolar injection of 0.2 ml of Dextran marked with a radiomarker (technetium – Tc 99 m) with activity of 0.4 mCi. Thus, the non-palpable lesion as well as the sentinel lymph node were identified through intra-operative monitoring with the gamma –probe.^11^

In all patients, we used a model XS wound retractor.^9^ All dissections were performed with a monopolar electric scalpel. During the conservative surgery, only one periareolar incision affecting half the areola was made, or a single incision in the mammary sulcus was made, measuring about 4 to 7 cm long for an areola measuring less than 3 cm in diameter. After the incision was made a peri-incisional dermo-cutaneous flap was made to introduce the retractor.^12^ It is important to highlight that the wound retractor offered protection, kept the surgical incision open constantly, and exposed the surgical field in a much better way compared with the usual retractors. Through only one incision and resting on the wound retractor, longer retractors with light and smoke evaporator are placed. 

**Fig. 1:**
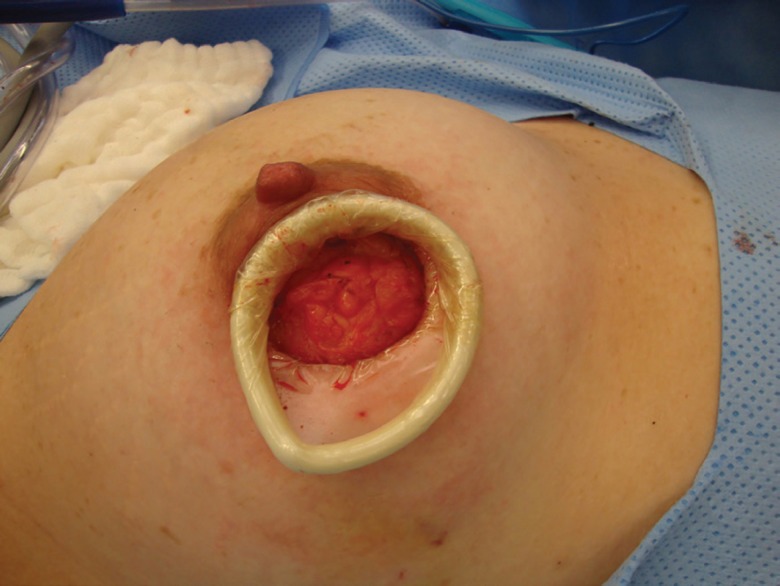
Peri areolar approach

**Fig. 2:**
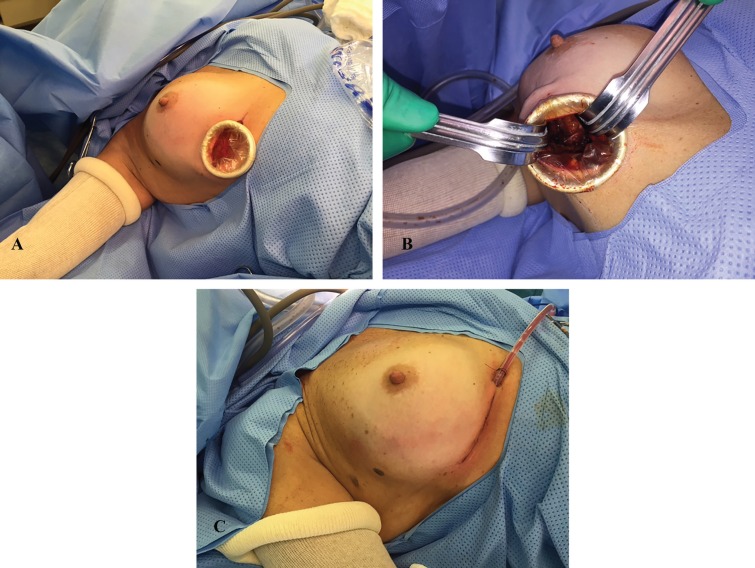
Inframammary sulcus approach. A: conservative surgery tumor on the intersection of lateral quadrants. B: Sentinel lymph node dissection. C: Final aspect

Thus the visualization of the mammary gland was very good because the incision was kept opened by the wound retractor and the long retractors. Next the dissection of cutaneous flap was performed through the superficial fascia, by detaching the glandular tissue until the tumoral area to be removed is reached. After the tumoral area was identified, the lesion and surrounding tissue were dissected, then detached from the deep fascia and removed. After the lesion was removed with the proper margin, the mammary gland was detached through the deep fascia until the axillary region was reached. At this point the sentinel lymph node was identified and removed and if necessary the other axillary lymph nodes were dissected (Figure 1 and 2). 

**Fig. 3:**
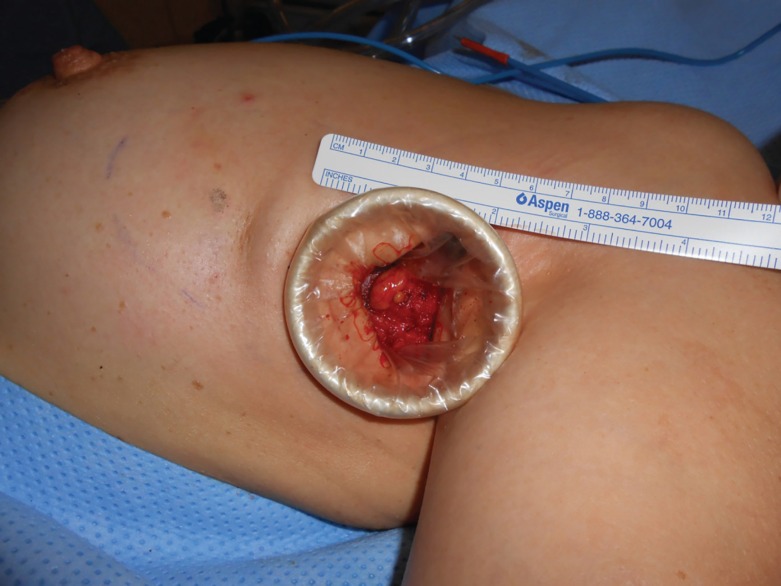
Axillary incision

In the case of the exclusive axillary approach, after incision, the wound retractor was inserted, followed by the retractor to facilitate the lymph node dissection (Figure 3). For nipple sparing, adenectomies**, **only one incision was made in the inframammary sulcus measuring between 9 to 12 cm long to remove the mammary gland and the sentinel lymph node or axillary lymph node dissection. A bigger wound retractor was inserted through this opening. Next an upper flap was made through the dissection of the superficial fascia. Later, through the deep fascia dissection, we displaced the mammary gland from the lower musculature. After having the breast totally superficially and deeply detached, we delimited the superior, lateral and medial edges and removed it. Through the cavity created with mammary gland removal and using retractors with light and smoke evaporator, the axillary region was approached. 

**Fig. 4:**
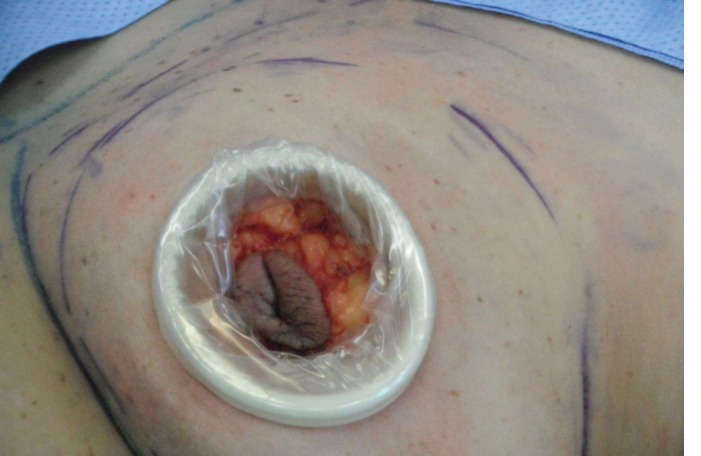
Skin sparing mastectomy

**Fig. 5:**
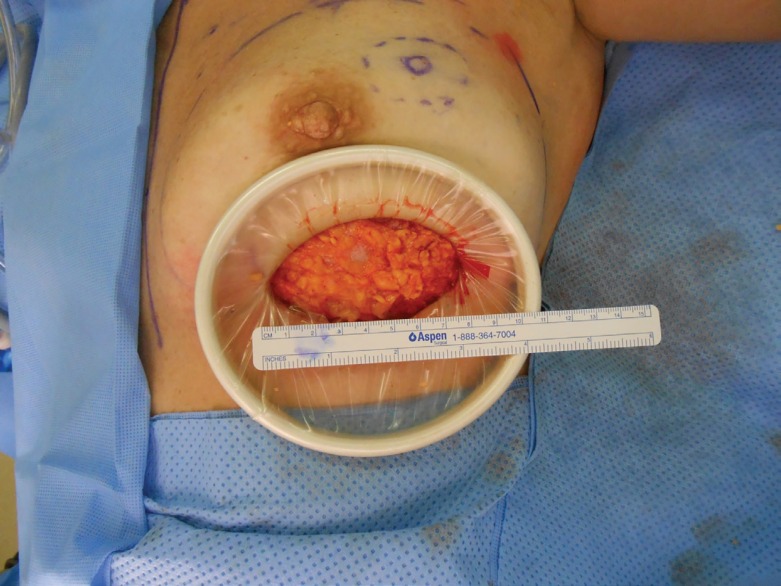
Nipple sparing mastectomy with incision in the infra mammary sulcus.

In cases of adenectomy with APC removal, only one circular incision was used (periareolar) with a variable size according to the areola size. Then a wound retractor was inserted where the APC was and the same dissection type as described above was performed. The patients who underwent adenectomy had breast reconstruction performed with an expander or definitive prosthesis depending on the case. At the end of the surgery, a vacuum drain proportional in size to the breast was inserted through a small incision (Figure 4 and 5). All resected material was sent to the pathologist, who during the intra-operative period verified the edges and analyzed the removed lymph node(s).

## RESULTS

We performed 31 surgical procedures using the single-incision technique: prime incision and modified moving window. There were 30 patients with primary breast cancer diagnosis stages I and II and 1 with loco-regional recurrence. We performed conservative surgery in 23, adenectomies in 7, and an axillary incision for level III interpectoral lymphadenectomy in 1. Among 23 patients who received the conservative surgery, the tumor was in the supero lateral quadrant in 8, the infero medial quadrant in 2, the supero medial quadrant in 4, the infero lateral quadrant in 2, the intersection of superior quadrants in 3, the intersection of inferior quadrants in 1, the intersection of medial quadrants in 1, and in the lateral quadrants in 2 (Figure 1).

Among the 7 patients who received adenectomy, the tumor was next to or behind the areola in 2; for the other 5 patients who had nipple-sparing mastectomy, the tumor volume to breast ratio was insufficient to permit conservative surgery (Figure 2). In 1 case, a single axillary incision was performed; this patient had a nipple-sparing mastectomy (stage II) 3 years before the study. She developed a loco-regional recurrence with lymph nodes in the interpectoral chain at stage III. It is suspected that all of them absorbed fluoride from axillary ultrasonography and positron emission computed tomography (Figure 3). All patients had surgery with only one incision as described above.

The mean patient age was 50.7 years. The most frequent histological type was infiltrating ductal carcinoma (80.6% of cases). The median tumor diameter was 1.63 cm. The surgical time was not longer than the usual time for these kinds of surgeries. The amount of intra-operative bleeding was within normal limits for these kinds of surgeries (conservative or adenectomies). No complication with regard to the fulfillment of the oncological surgical objectives or in the scarring process occurred. Median post-operative follow-up was 7.5 months (range, 1 to 16 months). Aesthetic results were as expected, and patients were very pleased that their breast was reconstructed with only one scar.

## DISCUSSION

The conservative surgery for breast cancer treatment has provided an excellent local control and generally speaking a great cosmetic result; however, if the aesthetic result is not satisfactory, the objective of the oncological surgical treatment may be blurred in the patient’s perception. Thus, besides providing a feasible oncological result, an excellent cosmetic result will avoid problems of personal dissatisfaction and sexual identity. The original moving window technique described by Noguchi and Onikuchi meets the criteria for conservative surgery or mastectomy with the removal of APC (skin-sparing mastectomy).^8^^,^^9^

We performed this surgery according to the original authors’ description; however, we removed the imposed conditions. We abolished the need for areolar-papillary complex mobility. The need for an adequate mammary volume is no longer a consideration. The indication for wound retractor use has been broadened thus allowing us to perform surgeries with only one incision. It may look simple at first sight; however, this technique requires training, learning, skills, and patience because the surgeon will have to operate through a smaller area than usual. For this surgical procedure it is essential to use long surgical instruments such as a wound retractor with light and smoke retractor, and an elongated electric scalpel. 

This study has shown that the prime incision and modified moving window techniques resulted in only one small scar and provided feasible breast cancer treatment with excellent acceptance and satisfaction by the patients. The prime incision and modified moving window techniques described above were shown to be feasible, with appropriate surgical access, and provided an excellent cosmetic result without an increase in complications.
